# Effect of Regular Aerobic Activity in Young Healthy Athletes on Profile of Endothelial Function and Platelet Activity

**DOI:** 10.1155/2017/8715909

**Published:** 2017-05-29

**Authors:** Katarzyna Podgórska, Arkadiusz Derkacz, Ewa Szahidewicz-Krupska, Jakub Jasiczek, Piotr Dobrowolski, Aneta Radziwon-Balicka, Robert Skomro, Andrzej Szuba, Grzegorz Mazur, Adrian Doroszko

**Affiliations:** ^1^Wrovasc-Integrated Cardiovascular Centre, Provincial Specialized Hospital, Kamieńskiego 73a, 51-124 Wroclaw, Poland; ^2^Department of Internal Medicine, Occupational Diseases and Hypertension, Wroclaw Medical University, Borowska 213, 50-556 Wroclaw, Poland; ^3^Department of Congenital Heart Diseases, Institute of Cardiology, Warsaw, Poland; ^4^Glostrup Research Institute, Glostrup, Denmark; ^5^Division of Respiratory, Critical Care and Sleep Medicine, Department of Medicine, University of Saskatchewan, Saskatoon, SK, Canada

## Abstract

The aim of the study was to assess the impact of regular professional sports activity on the endothelial and platelet function in young men. The studied group were 79 young men (18–40 y, 25 athletes and 54 without any regular physical activity). The nitric oxide (NO) metabolic pathway intermediates, oxidative stress markers, mediators of inflammation, and platelet aggregation were measured. Flow mediated dilation (FMD) was studied before and after intravenous 16,0 g L-arginine infusion, which was repeated after oral administration of acetylsalicylic acid (ASA-75 mg/day) for 4 days. Both groups had similar demographic characteristics. In the athletes, there was significantly higher hsCRP level, better serum lipid profile, and lower pulse pressure. Greater baseline FMD in athletes and in response to L-arginine disappeared following ASA treatment. There were no differences in the levels of the NO pathway metabolites. The control group was characterized by higher PAI-1 following ASA treatment and sICAM-1 both at baseline and after ASA, but no differences in MDA and 6-keto-PGF-1 alpha and platelet aggregation were noted. Regular professional physical activity modulates endothelial but not platelet function and may thus exert an effect on overall cardiovascular risk.

## 1. Introduction

Endothelial cells play a pivotal role in the maintenance of cardiovascular homeostasis [1]. Endothelial dysfunction has been demonstrated to play an important role in pathogenesis of atherosclerosis [2, 3].

Nonpharmacological methods, in addition to conventional pharmacological intervention, are postulated to play an important role in modulating the profile of endothelial function [2, 4].

Endothelial cells activity is strictly associated with the platelet function. Under pathological condition, endothelial dysfunction (ED) enhances endothelial interactions with platelets [5]. Regular physical activity increases blood flow and shear stress leading to augmented bioavailability of nitric oxide (NO), which is associated with its positive effects on endothelial function [6]. The increase in blood flow and changes in hemodynamics that occur during acute exercise may provide a stimulus for both, acute and chronic changes in vascular function.

Intensive training, especially associated with some sports disciplines, may excessively increase platelet aggregation promoting thus the risk of thrombotic events. Some literature data points at marathon and triathlon as disciplines of particular risk of thrombotic events [7]. While improvement of endothelial function is reflected by increased NO bioavailability, the exact mechanisms underlying this phenomenon remain unclear. Similarly, optimal training schedules, possible sequential changes, and the duration of beneficial effects of the activity under various conditions also remain largely unresolved [8]. Appropriate exercise training is a clinically proven, primary intervention that delays and in many cases prevents the health burdens associated with cardiovascular disorders, including type 2 diabetes and its components (metabolic syndrome, insulin resistance, and hyperinsulinemia), hypertension, and—as a result—the onset and progression of atherosclerosis [9].

Hence, the aim to this study was to assess the impact of a regular professional sports activity on the endothelial and platelet function in young men.

## 2. Material and Methods

All experiments were conducted and approved in accordance with the guidelines of the local Bioethics Committee and adhered to the principles of the Declaration of Helsinki and Title 45, U.S. Code of Federal Regulations, Part 46, Protection of Human Subjects (revised November 13, 2001, effective December 13, 2001) and all the patients enrolled had signed the informed consent to participate in the study.

### 2.1. Material

**Figure 1:**
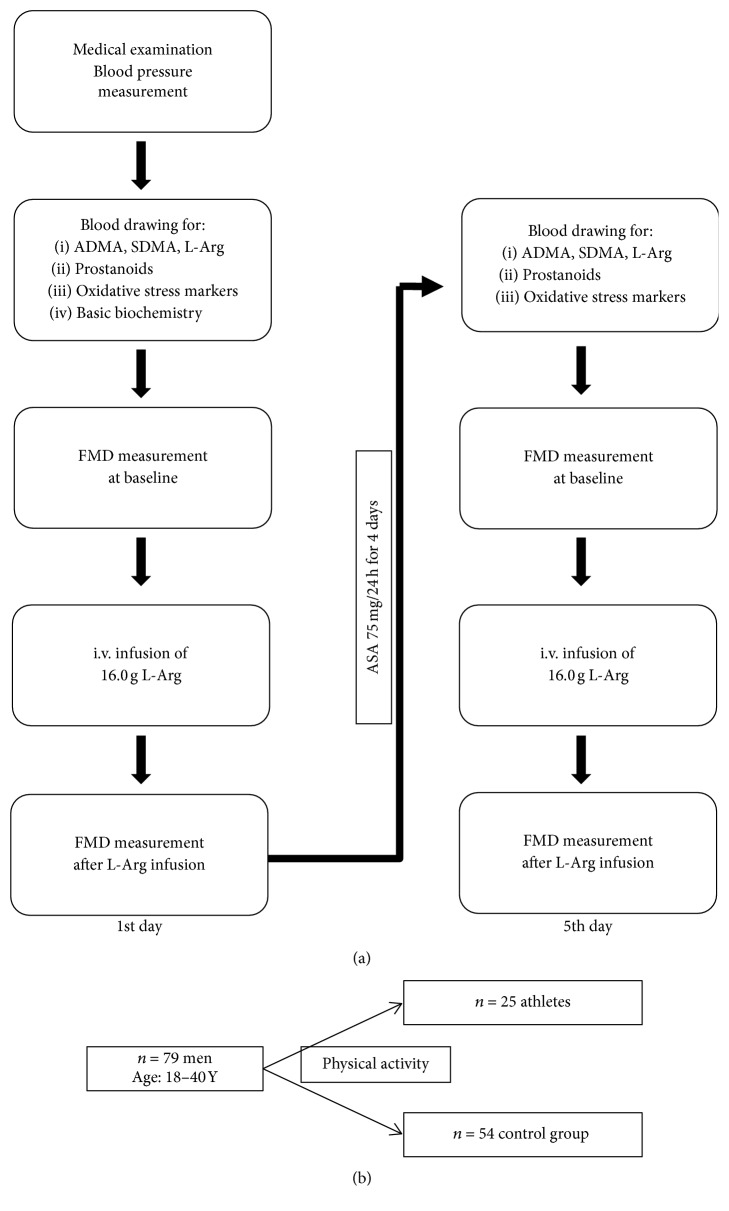
(a) A scheme of the study protocol. (b) Schematic subjects' categorization.

Healthy male volunteers (*n* = 79, mean age 18–40 years) were recruited to participate in this study. They were divided into two groups (Figure 1(b)):

- Subjects with regular sports activity (professional athletes group)
- Subjects with no sports activity (control group)

The investigated group included players from football and handball team. Their training was composed of 90 minutes of daily routine of aerobic and nonaerobic workout and games on weekends.

The second group included subjects without regular activity.

The recruited subjects were healthy, normotensive individuals. Exclusion criteria for the study were hypertension, coexisting diabetes mellitus, chronic inflammatory diseases, infections, malignancies, mental disorders, any pharmacological treatment, or allergy to nonsteroidal anti-inflammatory drugs.

### 2.2. Study Protocol

A scheme of the study protocol is shown in Figure 1(a). Each subject was given a medical examination. At 7:30–8:30 a.m. on the day of the medical exam, fasting blood was drawn from the antecubital vein. Blood was collected to the Sarstedt blood collecting system (S-Monovette®, Sarstedt, Germany). In order to avoid platelet activation, the blood was drawn without venous stasis. The following parameters were measured:

- Whole blood aggregation analysis.
- Metabolites of the NO pathway (asymmetrical dimethylarginine (ADMA), symmetrical dimethylarginine (SDMA), and L-arginine).
- Prostanoids levels (thromboxane B_2_ (TXB_2_) and 6-keto-prostaglandin F-1 alpha (6-keto-PGF-1 alpha)).
- The levels of markers of endothelial and platelet activation (soluble intercellular adhesion molecule-1 (sICAM-1), soluble vascular cell adhesion molecule-1 (sVCAM-1), vascular endothelial growth factor (VEGF), Serpin E1 = plasminogen activator inhibitor-1 (PAI-1), sE-Selectin, and sP-Selectin).
- Markers of oxidative stress (malondialdehyde (MDA) and thiol index, the reduced to oxidized glutathione ratio (GSH/GSSG)).
- Concentrations of total cholesterol (TCh), LDL (low-density lipoprotein) and HDL (high-density lipoprotein), triglycerides (TG), serum creatinine, glucose, hsCRP (high-sensitivity C-reactive protein), K^+^ (potassium ions), and uric acid.

For aggregation testing, whole blood with hirudin as anticoagulant was used. To obtain plasma, blood was collected in Sarstedt's S-Monovette (1,6 mg-EDTA/ml blood) tube and within 30 minutes following the collection was centrifuged at 1000 ×g for 15 min. at 4°C and next stored at −20°C until analysis of ADMA, SDMA, L-arginine levels, Serpin E1, sP-Selectin, and markers of oxidative stress. For assessment of the thiol index, whole blood with EDTA as anticoagulant was used. Serum for determination of VEGF, sICAM-1, sVCAM-1, sE-Selectin, and standard biochemical analysis was drawn to a tube with beads coated with a clotting activator (silicate). The samples were allowed to clot for 20–30 min. and were centrifuged at 1000 ×g for 15 min. at 4°C and stored at −20°C until analysis. To obtain plasma for determination of 6-keto-PGF-1 *α* and TXB_2_, whole blood was drawn into the sodium citrate tubes containing 500 *μ*l 5 mM crystalline acetylsalicylic acid (Sigma Aldrich®, USA) in 0,9% NaCl newly prepared to inhibit ex vivo platelet activity and within 30 minutes after collecting was centrifuged at 1000 ×g for 15 min. at 4°C and stored at −80°C until analysis.

After the blood had been drawn, the vasodilatory reactivity of the brachial artery after reversible ischemia—the FMD (flow mediated dilation)—was measured to diagnose ED. The cut-off point for ED was established as <8% change in the diameter of brachial artery in response to reactive hyperemia compared with the baseline value. Following the baseline FMD testing, the subjects were administered 16.0 g of L-arginine (Fresenius®, Germany) intravenously, followed by a second FMD measurement.

After being tested, all subjects were given 75 mg of acetylsalicylic acid (ASA) orally (Polocard 75 mg, Polpharma Co., Poland) daily for 4 days, after which all the procedures were repeated.

### 2.3. Aggregometry

Whole blood aggregation was measured using an impedance aggregometer (Multiplate® analyzer, Dynabyte Medical, Germany). During the analysis, the sample-reagent mixture was stirred with a discardable PTFE (polytetrafluoroethylene) coated magnetic stirrer (800 U/min). Preheated to 37°C, saline (300 *μ*l) was placed into the test cells with addition of anticoagulated whole blood (300 *μ*l) before or following the treatment with ASA. After 3 minutes of incubation and stirring at 37°C, the measurements were started by adding 20 *μ*l of the appropriate agonist (arachidonic acid to 0,5 mM of final concentration and ADP to 6,5 *μ*M of final concentration). The impedance change by the adhesion and aggregation of platelets on the electrode wires was continuously detected. The mean values of the two determinations are expressed in arbitrary units (AU).

### 2.4. Flow Mediated Dilation (FMD) Measurement

Ultrasound assessment of FMD in response to reactive hyperemia was performed according to the method described by Celermajer et al. [10] using the SSD 5500 duplex ultrasound machine (ALOKA, Japan) with a 7–14-MHz linear array transducer and the probe holder MP-AH 0001 (ALOKA, Japan) for immobilization of the arm/forearm. This was done in order to improve the accuracy of FMD measurement.

### 2.5. Measurement of the Markers of Nitric Oxide Metabolic Pathway and of Oxidative Stress

Plasma concentrations of L-arginine, ADMA, and SDMA were measured by a high-performance liquid chromatography (HPLC), and precolumn derivatization was measured with* o*-phthaldialdehyde (OPA) by a previously published method [11, 12].

MDA levels were assessed with a lipoperoxidation marker using a colorimetric assay BIOXYTECH® LPO-586™ (OxisResearch™, OXIS International Inc., USA). The thiol index, the reduced to oxidized glutathione ratio, was measured using a colorimetric assay BIOXYTECH GSH/GSSG-412™ (OxisResearch, OXIS International Inc., USA).

### 2.6. Measurement of Endothelial and Platelet Activation Markers

Plasma concentrations of sP-Selectin/CD62-P, Serpin E1/PAI-1, and serum concentrations of sE-Selectin/CD62E, sICAM-1/CD54, sVCAM-1/CD106, and VEGF were measured using commercial ELISA kits (R&D Systems® Europe, Ltd., UK) according to the manufacturer's instructions.

Plasma concentrations of prostanoids (6-keto-PGF-1 alpha as a marker of prostacyclin synthesis and TXB_2_ reflecting thromboxane formation) were measured using commercial EIA kits (Enzo Life Sciences GmbH, Germany) according to the manufacturer's instructions.

Carotid-femoral, carotid-radial, and carotid-dorsal pedis pulse wave velocities (PWV) were assessed using automatic Complior SP device (s/n 200901-000612, Artech Medical, France) and dedicated software (ver. 1.3.0). PWV (m/s) was calculated by dividing the distance between arteries by the time shift (transit time). After blood pressure measuring, four piezoelectric Complior transducers were placed on skin over the carotid, radial, femoral, and dorsalis pedis artery. After acceptable signal strength and quality had been obtained, pulse wave data was acquired and computer calculation was performed.

### 2.7. Other Biochemical Analyses

Concentrations of TCh, LDL, and HDL fractions and triglycerides, serum creatinine, fasting plasma glucose, hsCRP, K^+^, and uric acid were measured by standard laboratory assays.

### 2.8. Statistical Analysis

Data is expressed as the mean ± SEM (standard error of the mean). The differences between two continuous parameters were assessed using Mann–Whitney* U* test or Student's* t*-test, followed by a Shapiro-Wilk test and Levene's test as appropriate. All calculations were made using Statistica 10.0 (StatSoft®, USA).

## 3. Results

### 3.1. Baseline Demographic Characteristics

Both subgroups had a very similar demographic characteristics and pulse wave velocity reflecting arterial stiffness. Lipid profile differed favoring the group of athletes (total cholesterol and LDL serum level were lower, and HDL level was higher in this group). The pulse pressure was higher in the control group. None of the recruited patients was treated with any drug nor suffered from any chronic disease (Table 1).

A positive correlation between SBP and WHR in the control group (*R* = 0,47; *p* < 0,05) was shown, whereas similar correlation between SBP but with BMI in the athletes was demonstrated. What is more, in the controls, a positive correlation between SBP and triglycerides levels was observed (*R* = 0,43; *p* < 0,05). Interestingly, a high positive correlation between WHR and triglycerides levels in athletes was present (*R* = 0,71; *p* < 0,005).

### 3.2. Endothelial Vasodilative Function

Baseline FMD was significantly lower in nonactive patients, compared with athletes group. The difference was maintained following intravenous infusion of L-arginine; however, it disappeared following the treatment with ASA and after both L-arginine and the ASA treatment (Table 2).

### 3.3. Markers of Oxidative Stress and Nitric Oxide Metabolic Pathway

No significant differences were present in levels of ADMA, SDMA, and L-Arg (Table 2).

**Figure 2:**
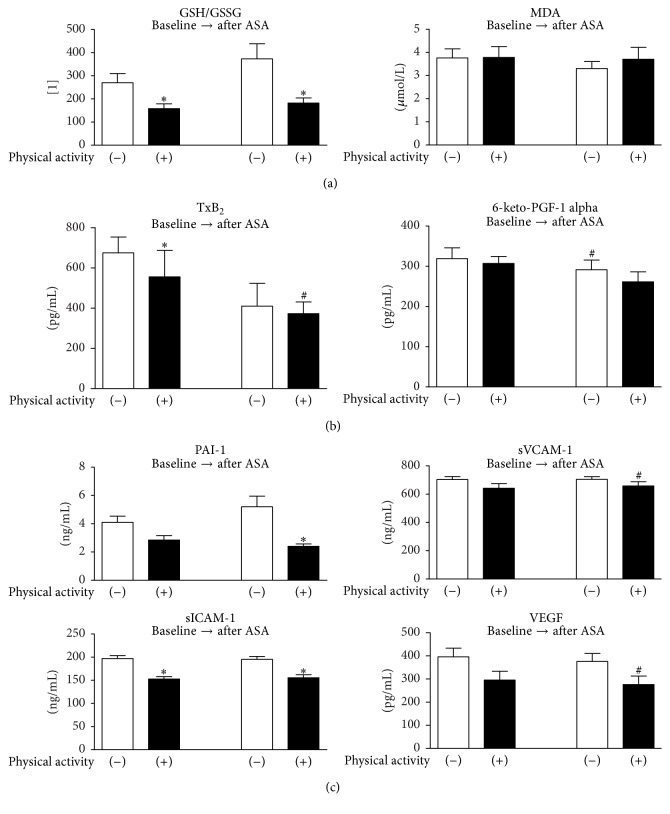
(a) Comparison of the oxidative stress markers in the subgroups at particular steps of the study protocol. (b) Comparison of the prostanoids levels in the subgroups at particular steps of the study protocol. (c) Comparison of the endothelial activation markers in the subgroups at particular steps of the study protocol. GSH/GSSG: thiol index, the reduced to oxidized glutathione ratio, MDA: malondialdehyde (lipid peroxidation marker), TXB_2_: thromboxane B_2_, 6-keto-PGF-1 alpha: 6-keto-prostaglandin F-1 alpha (a metabolite of prostacyclin, PGI_2_), PAI-1: plasminogen activator inhibitor-1, sVCAM-1: soluble vascular cell adhesion molecule-1, sICAM-1: soluble intercellular adhesion molecule-1, and VEGF: vascular endothelial growth factor. ^*∗*^*p* < 0,05 versus control. ^#^*p* < 0,05 versus the same group at baseline.

No significant differences appeared regarding MDA level, when comparing both groups. The thiol index was significantly higher in control group both, before and after ASA treatment (Figure 2(a)).

### 3.4. Markers of Endothelial and Platelet Activation

In the control group, there were significantly higher levels of 6-keto-PGF-1 alpha before the ASA treatment. No such changes were present when comparing both groups (Figure 2(b)).

Plasma levels of TXB_2_ before ASA treatment were higher in control group. The difference disappeared after ASA treatment (Figure 2(b)).

After ASA treatment, there were significant differences in PAI-1 levels (Figure 2(c)).

Higher concentration of sICAM-1 was present in the control group before and after ASA treatment (Figure 2(c)).

sVCAM-1 levels were significantly higher after ASA treatment in the athletes group. Such difference did not appear in other sVCAM-1 measurements (Figure 2(c)).

In the athletes group, the VEGF level was higher before ASA treatment. There were no differences when both groups were compared (Figure 2(c)).

No significant differences between groups or within the same group at particular steps of the study protocol were observed in the level of sP-Selectin and sE-Selectin.

### 3.5. Platelet Aggregation

No significant differences in platelet aggregation between groups in response to the ADP and arachidonic acid at baseline and following treatment with acetylsalicylic acid were observed. The decrease in arachidonic acid-induced aggregation, reflecting aspirin sensitivity, was similar in both groups (Table 3).

## 4. Discussion

### 4.1. Major Finding

This study demonstrates that professional physical activity exerts beneficial effects on endothelial function, assessed by measuring its vasodilative as well as inflammatory activity. However, the increase of platelet aggregation was not confirmed. Considering groups homogeneity regarding demographic characteristics, observed differences in serum lipid profile may be found due to the physical activity which differs in both groups.

### 4.2. Overall Cardiovascular Risk

The results of our study show that the mean pulse pressure value was significantly higher in the control group. Furthermore, there was a relationship between the waist to hips ratio (WHR) and systolic blood pressure (SBP) in control group, whereas in athletes systolic blood pressure relates to the body mass index (BMI). This fact may indicate different distribution of adipose tissue in control group. Thus, prospective studies are needed in order to analyze future risk for cardiovascular events in the control group. Blood pressure was rising with an increase in the levels of nonbeneficial lipid fractions (TG, total cholesterol) in both groups, which might confirm the relationship between decreased physical activity and metabolic syndrome characterized, that is, by impaired lipid profile. Numerous studies have confirmed the positive effect of regular physical activity on the clearance of triglycerides and on increase in the HDL-cholesterol fraction [13, 14]. However, less pronounced effect was noted, when changes in the concentrations of LDL-cholesterol were analyzed [15, 16].

In the present study, we observed significantly lower C-R PWV (carotid-radial PWV) and C-D PWV (carotid-dorsal pedis PWV) values but not C-F PWV (carotid-femoral PWV) in athletes group, which suggests that vascular destiffening effect of regular exercise is more prominent in the peripheral vasculature. This finding is consistent with a recently published meta-analysis, which shows that the effect of aerobic exercise on arterial stiffness tended to be greater in peripheral rather than in central vasculature [17].

### 4.3. Endothelial and Platelet Function

Greater FMD values observed in athletes group were not due to increased NO biosynthesis. Hence, the differences in endothelial vasodilative function could result from increased NO degradation. Moreover, the differences in thiol index could point at NO scavenging related to reactive oxygen species. Intravenous infusion of L-arginine enhances the differences in FMD and limits oxidative stress expressed by MDA levels, which confirms anti-inflammatory properties of NO postulated by other authors [18, 19].

The study protocol was aiming at the explanation whether lower FMD values reflecting the nitric oxide bioavailability were due to a decreased nitric oxide synthesis or its increased degradation [12]. Baseline differences in the levels of L-arginine (a substrate for endothelial nitric oxide synthase, eNOS) and ADMA (a competitive eNOS inhibitor) would reflect a different nitric oxide synthesis. On the other hand, lack of differences in the intermediates mentioned above combined with the presence of differences in the oxidative stress markers points at increased nitric oxide degradation by free oxygen radicals. The reactive oxygen species could partially derive from inflammatory reactions, which are limited by the cyclooxygenase inhibition using ASA at doses that also limit platelet aggregation, thus pointing at beneficial action of ASA not only in clinically relevant atherosclerosis, but also at early stages of clinically latent endothelial dysfunction.

Lower thiol index observed in the athletes indicates lower antioxidative capacity in this group and lower levels of inflammatory markers (sICAM1, sVCAM1, E-Selectin, and PAI-1) could be due to positive anti-inflammatory effect of regular physical training. Nonetheless, the athletes were characterized by a significantly higher CRP level. Some studies relate this observation to traumatism of high intense training [20]. Available clinical studies are inconclusive in this matter [21, 22]. On the other hand, higher sICAM1 level in control group may be related to the magnitude of systemic inflammatory processes, which may reflect acceleration of atherogenesis that might be abolished by regular training.

Our finding does not confirm the results presented in a recent meta-analysis [23], which indicated that greater FMD values in athletes were observed in master athletes but not in subgroup of young athletes. The population investigated in our study constituted young athletes at age of less than 40 years.

The results of this study have shown lower levels of VEGF in professional athletes, which reflects lower angiogenic potential. This data confirms the results of studies by other authors, which have shown the correlation between VEGF levels and both, the duration and intensity of regular training [24]. Initial increase in VEGF following high intensity training was associated with inflammatory response resulting from the damage to muscle fibers [25]. The regularity of training is associated with decreased angiogenic potential. In the athletes group, the long lasting and repetitive training, due to hypoxia episodes, could result in formation of collateral circulation leading in turn to an improvement of perfusion, which finally resulted in decreased angiogenic potential. This observation might be very interesting when the oncogenesis processes are considered.

The relations between PAI-1 and sICAM1, characterizing inflammatory process, and P-Selectin reflecting platelet activation confirm that inflammation activates platelets. In our study, there were no differences regarding parameters of platelet aggregation and activity.

## 5. Limitations

The experiment was limited by the number of professional athletes and women were not enrolled in the study. The chosen parameters are only few of all possible cardiovascular risk factors, but they constitute a base for research of high rate physical activity influence on endothelial function. The platelet activity was measured only as ADP aggregation, P-Selectin, and PAI-1 levels. Hence, more tests, such as thrombin or collagen aggregation, should be performed in order to analyze fully the effect of professional sport training on platelet function.

To summarize, the results of this study have confirmed positive modulating role of regular professional physical activity on phenotype of endothelial function. Furthermore, beneficial effect on the inflammatory reactions has also been observed.

## 6. Conclusions

The regular physical activity lowers the cardiovascular risk through modification of inflammation, endothelial vasodilation, and lipid profile. There was no influence on platelet aggregation.

*What Are the New Findings from the Study*

- Endothelial dysfunction in physically nonactive men results not from decreased nitric oxide synthesis but its increased degradation.
- Professional physical activity does not significantly affect platelet function.
- Lack of physical activity in young healthy men is associated with endothelial dysfunction.

## Figures and Tables

**Table 1:** Baseline demographic characteristics of analyzed groups.

Parameter (mean ± SEM)	Athletes *N* = 25	Control *N* = 54	*p*
Age [years]	23,52 ± 0,37	24,58 ± 0,55	0,07
HR [bpm]	69,00 ± 2,43	72,08 ± 1,94	0,21
SBP [mmHg]	122,91 ± 1,94	126,78 ± 2,57	0,07
DBP [mmHg]	81,45 ± 1,84	81,21 ± 1,89	1,00
MAP [mmHg]	95,27 ± 1,65	96,12 ± 1,96	0,55
PP [mmHg]	41,45 ± 1,89	46,40 ± 2,03	0,04
BMI [kg/m^2^]	25,82 ± 0,58	26,41 ± 0,66	0,57
WHR [1]	0,95 ± 0,0	0,938 ± 0,01	0,29
TCh [mg/dl]	161,04 ± 5,93	182,25 ± 6,95	0,01
HDL [mg/dl]	55,7 ± 2,83	48,73 ± 1,58	0,03
LDL [mg/dl]	82,28 ± 5,25	105,2 ± 5,70	0,006
TG [mg/dl]	112,36 ± 14,54	147,85 ± 12,46	0,07
hsCRP [mg/l]	3,52 ± 0,23	2,40 ± 0,37	0,003
Glucose [mg/dl]	84,66 ± 2,17	84,53 ± 1,81	0,68
Creatinine [mg/dl]	0,96 ± 0,02	0,96 ± 0,02	0,70
eGFR [ml/(min × 1,73 m^2^)]	147,48 ± 4,98	130,44 ± 8,19	0,07
Uric acid [mg/dl]	6,15 ± 0,26	6,18 ± 0,23	0,98
K^+^ [mmol/l]	3,98 ± 0,05	5,03 ± 0,05	0,10
Na^+^ [mmol/l]	142,65 ± 0,22	141,42 ± 0,32	0,02
Urea [mg/dl]	36,62 ± 1,41	31,4 ± 1,35	0,003
C-R PWV [m/s]	9,61 ± 0,36	10,69 ± 0,34	0,04
C-F PWV [m/s]	9,51 ± 0,24	9,90 ± 0,32	0,67
C-D PWV [m/s]	8,15 ± 0,19	8,91 ± 0,24	0,01

SBP: systolic blood pressure; DBP: diastolic blood pressure; MAP: mean blood pressure; PP: pulse pressure; BMI: body mass index; WHR: waist-hips ratio; TCh: total cholesterol; HDL: high-density lipoproteins; LDL: low-density lipoproteins; TG: triglycerides; eGFR: estimated glomerular filtration rate; hsCRP: high-sensitivity C-reactive protein; C-R PWV: carotid-radial pulse wave velocity, C-F PWV: carotid-femoral pulse wave velocity; C-D PWV: carotid-dorsal pedis pulse wave velocity.

**Table 2:** Flow mediated dilatation (FMD) and markers of the nitric oxide metabolic pathway in both groups.

Parameter (mean ± SEM)	Athletes *N* = 25	Control *N* = 54	*p*
FMD [%]			
Baseline	9,62 ± 0,70	8,05 ± 0,59	0,049
Baseline + L-Arg	11,74 ± 0,60	8,93 ± 0,67	0,01
	*p* = ns	*p* = ns	

FMD [%]			
ASA	9,20 ± 0,70	7,77 ± 0,82	ns
ASA + L-Arg	8,94 ± 0,56	7,86 ± 0,85	ns
	*p* = ns	*p* = ns	

L-arginine [*μ*mol/l]			
Baseline	60,33 ± 3,83	65,07 ± 2,47	ns
After ASA	68,37 ± 5,37	63,73 ± 2,73	ns
	*p* = ns	*p* = ns	

ADMA [*μ*mol/l]			
Baseline	0,42 ± 0,016	0,42 ± 0,01	ns
After ASA	0,42 ± 0,02	0,41 ± 0,01	ns
	*p* = ns	*p* = ns	

SDMA [*μ*mol/l]			
Baseline	0,46 ± 0,016	0,47 ± 0,02	ns
After ASA	0,47 ± 0,018	0,51 ± 0,03	ns
	*p* = ns	*p* = ns	

FMD: flow mediated dilation, ADMA: asymmetric dimethylarginine, L-Arg: L-arginine, ASA: acetylsalicylic acid, and SDMA: symmetric dimethylarginine.

**Table 3:** Platelet aggregation in both groups.

Parameter (mean ± SEM)	Athletes *N* = 25	Control *N* = 54	*p*
Arachidonic acid-induced aggregation [AU]			
Baseline	151,81 ± 7,72	147,11 ± 4,08	ns
After ASA	90,8 ± 11,47	73,37 ± 5,69	ns
	*p* = 0,002	*p* = 0,000	

ADP-induced aggregation [AU]			
Baseline	122,73 ± 7,39	118,39 ± 4,07	ns
After ASA	114,88 ± 8,48	106,63 ± 5,79	ns
	*p* = ns	*p* = ns	

## References

[1] Chesterman C. N. (1988). Vascular endothelium, haemostasis and thrombosis. *Blood Reviews*.

[2] Drexler H., Hornig B. (1999). Endothelial dysfunction in human disease. *Journal of Molecular and Cellular Cardiology*.

[3] Mombouli J.-V., Vanhoutte P. M. (1999). Endothelial dysfunction: from physiology to therapy. *Journal of Molecular and Cellular Cardiology*.

[4] Esposito K., Marfella R., Ciotola M. (2004). Effect of a mediterranean–style diet on endothelial dysfunction and markers of vascular inflammation in the metabolic syndrome: a randomized trial. *The Journal of the American Medical Association*.

[5] Shimada K. (1993). Molecular biology of endothelium–platelet interactions and thrombogenesis. *Japanese Journal of Clinical Medicine*.

[6] Di Francescomarino S., Sciartilli A., Di Valerio V., Di Baldassarre A., Gallina S. (2009). The effect of physical exercise on endothelial function. *Sports Medicine*.

[7] Hanke A. A., Staib A., Görlinger K., Perrey M., Dirkmann D., Kienbaum P. (2010). Whole blood coagulation and platelet activation in the athlete: a comparison of marathon, triathlon and long distance cycling. *European Journal of Medical Research*.

[8] Maiorana A., O'Driscoll G., Taylor R., Green D. (2003). Exercise and the nitric oxide vasodilator system. *Sports Medicine*.

[9] Bird S. R., Hawley J. A. (2012). Exercise and type 2 diabetes: new prescription for an old problem. *Maturitas*.

[10] Celermajer D. S., Sorensen K. E., Gooch V. M. (1992). Non-invasive detection of endothelial dysfunction in children and adults at risk of atherosclerosis. *The Lancet*.

[11] Teerlink T., Nijveldt R. J., De Jong S., Van Leeuwen P. A. M. (2002). Determination of arginine, asymmetric dimethylarginine, and symmetric dimethylarginine in human plasma and other biological samples by high–performance liquid chromatography. *Analytical Biochemistry*.

[12] Doroszko A., Szahidewicz-Krupska E., Janus A. (2015). Endothelial dysfunction in young healthy men is associated with aspirin resistance. *Vascular Pharmacology*.

[13] Cooper S. B., Dring K. J., Nevill M. E. (2016). High–intensity intermittent exercise: effect on young people's cardiometabolic health and cognition. *Current Sports Medicine Reports*.

[14] Gordon B., Chen S., Durstine J. L. (2014). The effects of exercise training on the traditional lipid profile and beyond. *Current Sports Medicine Reports*.

[15] Coelho D. F., Pereira-Lancha L. O., Chaves D. S. (2011). Effect of high–fat diets on body composition, lipid metabolism and insulin sensitivity, and the role of exercise on these parameters. *Brazilian Journal of Medical and Biological Research*.

[16] Sahlin K., Sallstedt E. K., Bishop D., Tonkonogi M. (2008). Turning down lipid oxidation during heavy exercise–what is the mechanism?. *Journal of Physiology and Pharmacology*.

[17] Ashor A. W., Lara J., Siervo M., Celis-Morales C., Mathers J. C. (2014). Effects of exercise modalities on arterial stiffness and wave reflection: a systematic review and meta–analysis of randomized controlled trials. *PLoS ONE*.

[18] Dusserre N., L'Heureux N., Bell K. S. (2004). PECAM-1 interacts with nitric oxide synthase in human endothelial cells: implication for flow-induced nitric oxide synthase activation. *Arteriosclerosis, Thrombosis, and Vascular Biology*.

[19] Wallace J. L., Ianaro A., Flannigan K. L., Cirino G. (2015). Gaseous mediators in resolution of inflammation. *Seminars in Immunology*.

[20] Chen T. C., Hsieh S. S. (2001). Effects of a 7-day eccentric training period on muscle damage and inflammation. *Medicine & Science in Sports & Exercise*.

[21] Kazeem A., Olubayo A., Ganiyu A. (2012). Plasma nitric oxide and acute phase proteins after moderate and prolonged exercises. *Iranian Journal of Basic Medical Sciences*.

[22] Andersson J., Jansson J.-H., Hellsten G., Nilsson T. K., Hallmans G., Boman K. (2010). Effects of heavy endurance physical exercise on inflammatory markers in non–athletes. *Atherosclerosis*.

[23] Montero D., Padilla J., Diaz-Cañestro C. (2014). Flow-mediated dilation in athletes: influence of aging. *Medicine and Science in Sports and Exercise*.

[24] Jensen L., Schjerling P., Hellsten Y. (2004). Regulation of VEGF and bFGF mRNA expression and other proliferative compounds in skeletal muscle cells. *Angiogenesis*.

[25] Czarkowska-Paczek B., Bartlomiejczyk I., Przybylski J. (2006). The serum levels of growth factors: PDGF, TGF-beta and VEGF are increased after strenuous physical exercise. *Journal of Physiology and Pharmacology*.

